# Allogenic Stem Cell Transplantation Abrogates Negative Impact on Outcome of AML Patients with KMT2A Partial Tandem Duplication

**DOI:** 10.3390/cancers13092272

**Published:** 2021-05-10

**Authors:** Gabriel Antherieu, Audrey Bidet, Sarah Huet, Sandrine Hayette, Marina Migeon, Lisa Boureau, Pierre Sujobert, Xavier Thomas, Hervé Ghesquières, Arnaud Pigneux, Mael Heiblig

**Affiliations:** 1 Department of Hematology, Lyon-Sud Hospital, Hospices Civils de Lyon, 69495 Pierre Bénite, France; gabriel.antherieu@protonmail.com (G.A.); xavier.thomas@chu-lyon.fr (X.T.); herve.ghesquieres@chu-lyon.fr (H.G.); 2 Hematology Biology, Molecular Hematology, Bordeaux University Hospital, 33600 Pessac, France; audrey.bidet@chu-bordeaux.fr (A.B.); marina.migeon@chu-bordeaux.fr (M.M.); lisa.boureau@chu-bordeaux.fr (L.B.); 3 Department of Molecular Hematology, Lyon-Sud Hospital, Hospices Civils de Lyon, 69495 Pierre Bénite, France; sarah.huet@chu-lyon.fr (S.H.); sandrine.hayette@chu-lyon.fr (S.H.); pierre.sujobert@chu-lyon.fr (P.S.); 4 Hematology and Cell Therapy, Bordeaux University Hospital, 33600 Pessac, France; arnaud.pigneux@chu-bordeaux.fr

**Keywords:** acute myeloid leukemia, KMT2A, transplantation

## Abstract

**Simple Summary:**

Acute myeloid leukemia refers to a large group of diseases that can be further defined by their genetic modifications. A specific alteration called *KMT2A-PTD* has been previously found in 5% of newly diagnosed acute myeloid leukemia cases. This work aimed to provide specific insight into outcomes of this specific disease and identify factors that may influence survival. Integrating *KMT2A-PTD* in ELN classification abrogates its predictive value on survival suggesting that this mutation may overcome other genomic marker effects. In patients receiving intensive chemotherapy, hematopoietic stem cell transplantation (HSCT) significantly improved the outcome compared to non-transplanted patients. In conclusion, our results emphasize that *KMT2A-PTD* should be considered as a potential adverse prognostic factor.

**Abstract:**

Recently, a new subset of acute myeloid leukemia (AML) presenting a direct partial tandem duplication (PTD) of the *KMT2A* gene was described. The consequences of this alteration in terms of outcome and response to treatment remain unclear. We analyzed retrospectively a cohort of *KMT2A-PTD*-mutated patients with newly diagnosed AML. With a median follow-up of 3.6 years, the median overall survival was 12.1 months. *KMT2A-PTD*-mutated patients were highly enriched in mutations affecting epigenetic actors and the *RTK/RAS* signaling pathway. Integrating *KMT2A-PTD* in ELN classification abrogates its predictive value on survival suggesting that this mutation may overcome other genomic marker effects. In patients receiving intensive chemotherapy, hematopoietic stem cell transplantation (HSCT) significantly improved the outcome compared to non-transplanted patients. In the multivariate analysis, only HSCT at any time in complete remission (HR = 2.35; *p* = 0.034) and *FLT3-ITD* status (HR = 0.29; *p* = 0.014) were independent variables associated with overall survival, whereas age was not. In conclusion, our results emphasize that *KMT2A-PTD* should be considered as a potential adverse prognostic factor. However, as *KMT2A-PTD*-mutated patients are usually considered an intermediate risk group, upfront HSCT should be considered in first CR due to the high relapse rate observed in this subset of patients.

## 1. Introduction

Due to a better biomolecular definition and recent approval of various targeted therapies, the overall prognosis of acute myeloid leukemia (AML) is moving toward tremendous improvement. Recent advances in the discovery of the genomic landscape of the disease, in the development of assays for genetic testing and for detecting minimal residual disease (MRD), have recently modified AML management [1,2,3]. Rearrangement involving the Lysine (K)-specific Methyltransferase 2A (*KMT2A*) gene located at 11q23, formerly known as the mixed lineage leukemia (*MLL*) gene, is found in approximatively 3% of de novo AML [4]. This abnormality is notoriously known to be associated with aggressive disease courses and poor prognosis [4,5,6]. As well as this rearrangement, *KMT2A* gene may show in-frame partial tandem duplication affecting the N-terminal region. According to the literature, this partial tandem duplication (PTD) is spanning exon 3 to 9, 3 to 10 or 3 to 11 [7,8], which corresponds to exon 2 to 8, to 9 or to 10 in NM_001197104 in order to comply with the frame. *KMT2A-PTD* seems to be related to epigenetic modifications resulting in abnormal Homebox (Hox) gene expression [8]. However, the pathogenic difference between rearranged *KMT2A* and *KMT2A-PTD* AML is rather unknown. Hinai et al. showed that *KMT2A-PTD* may induce overexpression of HOX-related genes in different ways than t(11q23)-related fusion proteins. The authors thus suggested that *KMT2A*-*PTD* induces leukemogenesis by mechanisms distinct from t(11q23) abnormalities involving *KMT2A* [9]. Converse to *KMT2A*-rearranged AML, *KMT2A-PTD* enhances self-renewal of hematopoietic stem cells by blocking the cell differentiation but is not sufficient by itself to induce overt leukemia. Indeed, Shnittger et al. showed that small clones of *KMT2A-PTD* could be found in healthy bone marrow samples without any signs of myeloid neoplasm, suggesting that other molecular or epigenetics events are necessary to induce overt leukemia [10].

In addition to *KMT2A* rearrangements, *KMT2A-PTD* have been reported in 3–10% of adult AML cases and are mutually exclusive to 11q23 rearrangements [7,8]. In a cohort of 986 adult AML patients, *KMT2-PTD* was found in 5% of cases, with some patients carrying concurrent *FLT3-ITD* mutations [4]. *KMT2A-PTD* event is usually associated with normal karyotype or +11. However, clinical implications of *KMT2A-PTD* on outcome also remain unclear with conflicting results [4,5,6,7]. In a cohort of 109 *KMT2A-PTD* patients treated intensively, Hinai et al. showed that *KMT2A-PTD* did not associate with outcome in AML. However, specific concurrent mutational subtypes such as *DNMT3A* and *NRAS* appear to carry poor prognostic value. Interestingly, Steudel et al. showed that *FLT3-ITD* and *KMT2A-PTD* have the same poor outcome when censored at the time of transplant, whether mutations co-occurred or not [4]. Finally, Haferlach et al. assessed that despite differences in the age of presentation and co-mutational burden, *KMT2-PTD* AML shared similar poor outcomes compared to *KMT2A*-rearranged AML. In this study, the authors also showed that the *IDH* specific molecular subgroup was associated with a reduced overall survival [10]. *KMT2A*-rearranged AML is considered to be an unfavorable prognosis within ELN 2017 classification [10,11,12]. However, *KMT2A-PTD* is not included in current AML prognostic classifications, and so, not considered as an independent factor for hematopoietic stem cell transplantation (HSCT) allocation.

In this study, we aimed to evaluate *KMT2A-PTD* impact on outcome in newly diagnosed AML patients according to treatment intensity and HSCT.

## 2. Patients and Methods

### 2.1. Patients

Seventy-nine patients (19–87 years old) treated in Lyon and Bordeaux, France, with newly diagnosed *KMT2A-PTD*-mutated AML (de novo or secondary) were included in this retrospective study, between November 2005 and August 2019. At diagnosis, blood and BM samples were examined for cytogenetic abnormalities and molecular markers (*NPM1, FLT3-ITD*) according to local procedures. Patients were assigned to risk groups according to the 2013 ELN classification, as *FLT3-ITD* ratio and next generation sequencing were not available for all patients at diagnosis. Cytogenetic analysis was performed according to the International System for Human Cytogenetic Nomenclature guidelines [13].

### 2.2. Outcome Parameters

CRc (CR + CRi + CRp) status was defined on BM aspirates with less than 5% of blasts recovery and classical hematological recovery characteristics. Overall survival (OS) was calculated from treatment assignment to death from any cause. Leukemia-free survival (LFS) was determined for responders from CRc until diseases relapse or death of any cause. Patients alive were censored for OS at last follow-up date, and patients in CR were censored for LFS at their last disease assessment. Survival analyses were not censored at the time of transplant

### 2.3. Treatments

Intensively treated patients received anthracycline and cytarabine-based induction chemotherapy regimen (*n* = 54). Patients achieving complete remission (CR) after one or two courses of induction were given consolidation chemotherapy with intermediate/high dose cytarabine. Low intensive chemotherapy group comprised patients treated frontline by low dose cytarabine (*n* = 9) or hypomethylating agents (*n* = 7). Policy with regard to blood product support, antibiotics and anti-fungal prophylaxis, and treatment of febrile neutropenia were determined according to established local practice [14]. The third study group (*n* = 10) comprised patients treated by best supportive care (BSC) associated if necessary, to the administration of hydroxyurea or 6-mercaptopurine in order to control proliferative white blood cell (WBC) count.

### 2.4. Statistical Analyses

Comparative descriptive statistics were used to characterize patients and their disease in their entirety. Continuous variables were reported as median ± standard deviation (SD) followed by a t test if the distribution was normal in both groups, if not as median, range (min–max) and inter-quartile range (IQR) with a Mann–Whitney test to compare groups. Discrete and qualitative variables were reported as count and percentage. Comparisons among groups were analyzed with a Pearson Chi-square test (with the Monte-Carlo method if any cell count was below 5). Probabilities of LFS and OS were estimated using the Kaplan–Meier method, and the log-rank rest evaluated differences between survival distributions. Univariate and multivariate analyses including baseline demographic, clinical and molecular features were studied thanks to Cox regressions. The statistical results were two-sided with a *p*-value < 0.05 considered statistically significant (XLSTAT^©^, 2020, Addinsoft, Paris, France).

## 3. Results

### 3.1. Patient Characteristics

The median age of the entire cohort was 66 years old (range 19–87). The patient characteristics are summarized in Table 1. Overall, most of the patients had a normal karyotype (65.3%). The most frequent recurrent cytogenetic abnormalities were +11 (3/72) and +8 (4/72). One patient harbored a t(8;21). Regarding 2013 ELN classification, 11.4%, 77.2% and 11.2% were favorable, intermediate I/II and unfavorable, respectively. Sixteen percent of patients were NPM1 and 20% were FLT3-ITD mutated. Among the 19 FLT3-ITD positive patients, 78.9% of them harbored a normal karyotype. No patients with FLT3-ITD mutation were NPM1 positive. Of the 79 KMT2A-PTD AML patients, 24% were considered as secondary, mostly after previously known myelodysplastic syndrome (10/20).

### 3.2. Overall Outcomes

**Figure 1:**
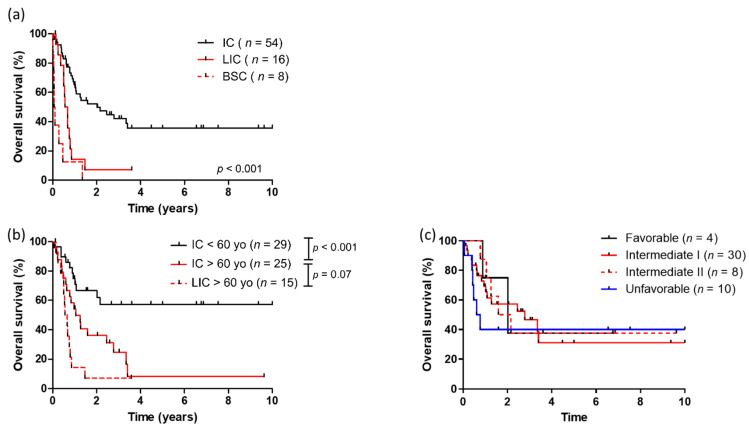
(**a**) Overall survival according to treatment intensity; (**b**) overall survival according to age and treatment intensity; (**c**) overall survival according to ELN 2013 classification in intensively treated patients. IC = intensive care, LIC = low intensive care, BSC = best supportive care.

The median follow-up time for the entire cohort was 3.6 years (1st quartile–3rd quartile: 2.6–6.86). In the intensively treated patients, the overall CRc rate was 64.5% (68.9% and 60% in patients younger and older than 60 years old, respectively). No CR was observed in those treated with low intensive regimens. When considering the whole cohort, the median overall survival (OS) was 12.1 months with an OS rate of 50.1% at 1 year and 30.9% at 3 years. The median leukemia free survival (LFS) was 9.5 months with an LFS rate of 43% and 27% at 1 and 3 years, respectively. Median OS for patients treated with intensive, semi-intensive and best supportive care was 24.4, 6.7 and 1 months, respectively (Figure 1a). Median LFS for the intensively treated group was 15.3 months compared to 6.7 months for those treated with low intensive regiments (*p* < 0.0001). In patients younger than 60 years old treated with intensive chemotherapy, median overall survival was not reached compared to 12.6 months in older ones (*p* < 0.001). In contrast, there was no statistical difference in OS in elderly patients treated with intensive or low intensive regimens (Figure 1b). When focusing on *KMT2A-PTD* patients treated with intensive chemotherapy (*n* = 52), ELN 2013 classification was not of statistical significance regarding prognosis (Figure 1c). When available (*n* = 44), similar observation was made regarding ELN 2017 classification.

### 3.3. Molecular Characteristics of KMT2A-PTD Patients and Their Impact on Intensively Treated Patients Outcome

**Figure 2:**
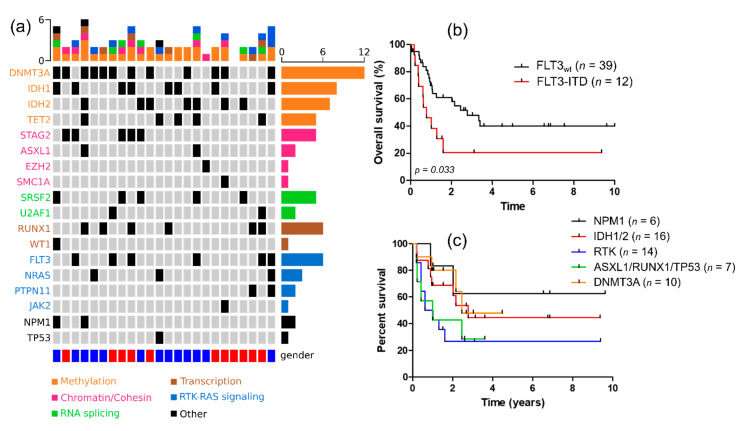
(**a**) Oncoplot (*n* = 24); (**b**) overall survival according to *FLT3-ITD* status; (**c**) overall survival according to molecular subgroups.

NGS was available for 24 intensively treated patients, of which 23 out of 24 had at least one recurrent genetic alteration. The number of detected mutations ranged from 0 to 6, with a median mutation number of 2.5. The most frequently observed mutations were those affecting DNA methylation (*DNMT3A, TET2, IDH1/2*), found in 91% (21/23) of cases. Receptor tyrosine kinase (RTK) and RAS pathways were mutated in about 44% of patients. Other pathways commonly found altered was the chromatin/cohesin complex (*ASXL1, STAG2, SMC1A, EZH2*) (39%), spliceosome (U2AF1, SRFS2) and transcription factors (*RUNX1, WT1*) in 39%, 30% and 30% of cases, respectively (Figure 2a). Regarding the impact of co-mutation on survival, *FLT3-ITD* was associated with an inferior outcome compared to wild-type ones (median OS: 9.36 vs. 33.4 months, *p* = 0.033) (Figure 2b). Results were similar regarding LFS (median OS: 6.48 vs. 25.9 months, *p* = 0.038). Median OS was not reached, 33.4 and 29.4 months for *NPM1-, IDH1/2-* and *DNMT3A*-mutated patients, respectively. In contrast, median OS was shorter for patients harboring RTK (*FLT3-ITD, NRAS, PTPN11)* and *TP53/ASXL1/RUNX1* mutations (9.6 and 11.9 months, respectively, *p* = 0.0475) (Figure 2c).

### 3.4. Impact of HSCT on Outcome

**Figure 3:**
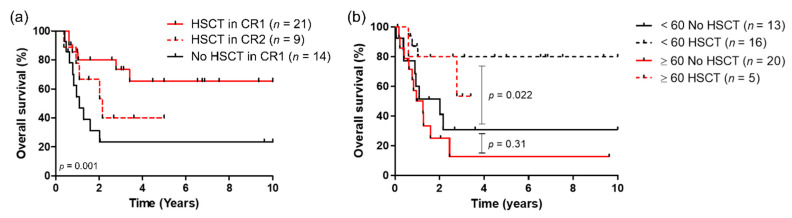
(**a**) Overall survival according to HSCT status; (**b**) overall survival according to age and HSCT in CR1 compared to patients who did not underwent HSCT at any time.

Overall, twenty-one patients underwent HSCT in first CRc (CR1) after reduced intensity conditioning (RIC) (*n* = 10) or myeloablative conditioning (*n* = 11). The median age of patients receiving HSCT was 56.5 years old compared to 67.2 for those who did not (*p* < 0.001). For patients aged over 60, there was no significant difference regarding median age between those receiving HSCT and those who did not (63.6 vs. 66.7, *p* = 0.12). Overall, treatment-related mortality was 14.6% (3/21). Patients receiving HSCT in CR1 had a better outcome compared to those who only received conventional consolidations (Figure 3a). Cumulative incidence of relapse in patients without HSCT in CR1 was 73.9% at 1 year. Of the 19 refractory patients that received salvage chemotherapy, nine were consolidated with HSCT in second CR with a median OS of 25.9 months. For patients receiving HSCT in CR1, median OS was not reached, no matter the age group. For those who did not undergo HSCT, median OS was 24.2 and 13.2 months in patients younger and older than 60 years old, respectively, with no statistical significance between age group in these settings (Figure 3b).

### 3.5. Univariate and Multivariate Analysis

In univariate analysis, only age, performance status, HSCT and *FLT3-ITD* status were significantly associated with overall survival. Results were similar for leukemia free survival with the exception of performance status (Table S1). In the multivariate analysis (including all variables statistically significant in the univariate analysis), only HSCT at any time in CR (HR = 2.35; *p* = 0.034) and *FLT3-ITD* status (HR = 0.29; *p* = 0.014) were independent variables associated with overall survival, whereas age was not (Table 2). Results were the same when using age as a continuous variable.

## 4. Discussion

To our knowledge, this study is one of the largest reported *KMT2A-PTD* cohorts emphasizing the impact of this rearrangement according to treatment intensity and HSCT. In real-life settings, patients harboring such abnormalities are characterized by a poor outcome. However, the independent prognosis value of *KMT2A-PTD* is still unclear with contradicting results reported in the literature so far [6,11]. This might be due to the relative low frequency of this rearrangement, especially in younger patients. Our results support that *KMT2A-PTD* might be more frequent in patients older than 60 years old. However, most of the clinical studies focusing on *KMT2A-PTD* are usually based on prospective clinical trial enrolling patients below the age of 65 years old [10,11]. Based on a large unselected and well-defined cohort of 956 patients, Shih et al. reported that the *KMT2A-PTD* mutant has a similar outcome than *KMT2A*-rearranged AML [6,15]. The same negative impact of *KMT2A-PTD* was found by Döhner et al. with a median PFS of 7.75 vs. 19 months [11]. These results are consistent with those found in pediatric settings in which *KMT2A-PTD* was associated with a poor prognosis [16]. In a cohort of patients with a median age of 55 years, Hinai et al. did not find any significant difference in terms of OS and LFS between mutated and unmutated *KMT2A-PTD* AML, with a much better median OS than previously described [9]. We show that the negative impact of this mutation might be overcome by HSCT. In numerous reported studies, very little information was given regarding therapy, and OS results were usually censored at the time of HSCT [6]. We found that the median OS of younger patients was clearly improved by transplantation. However, even if a trend was observed, it did not reach statistical significance in patients aged over 60 years old. Interestingly, younger patients who cannot achieve HSTC have similar outcome than older ones. Numerous prospective and retrospective studies have been necessary to confirm that intermediate *FLT3-ITD*-mutated patients truly benefit from matched HSCT in CR1 [17,18]. As *KMT2A-PTD* is less frequent than *FLT3-ITD* and tends to co-occur with *FLT3-ITD* and also cluster in the intermediate risk group, assessing HSCT’s true benefit on survival might be challenging.

By deciphering clonal architecture, Tan et al. showed that *KMT2A-PTD* was not a driver mutation and usually arises after *IDH2/1, DNMT3A, U2AF1* or *TET2* mutations. Interestingly, proliferative mutations such as *FLT3-ITD* or *RAS* mutations seemed to appear later and were largely subclonal [19]. These clinical observations are in line with the previously reported *KMT2A-PTD* mice model. Indeed, *KMT2A-PTD* expressing mice do not develop overt leukemia and usually need a second hit such as *FLT3-ITD* to induce an aggressive myeloproliferative phenotype. Conversely, K*MT2A*-rearanged AML are usually defined by low co-mutational burden and also complex karyotypes [12]. Despite affecting a similar gene, clonal evolution and leukemogenesis in these two entities might be rather different. As previously reported, we observed a high rate of epigenetic alterations such as *DNMT3A, IDH1/2* or *TET2* mutations which are usually lower in newly diagnosed AML [10]. We may emphasize that these latter genomic alterations might be the original AML driver events and *KMT2A-PTD* a secondary clonal event. In Hinai et al. study, concomitant mutation of *DNMT3A* was found to be associated with poor outcome [7]. We did not confirm this result that might have been biased by its high frequency and the size of our cohort. However, our work clearly corroborated the negative prognostic impact of co-occurring RTK/RAS pathway mutations as previously described [9]. As *KMT2A-PTD* seems to occur more frequently in elderly, its negative impact on survival might be overcome by other confounding factors and age-related therapeutic limitations. Finally, integrating *KMT2A-PTD* in ELN classification abrogate its predictive value on survival suggesting that this mutation may overcome other genomic markers effect, especially in younger patients. However, these results might be taken with cautions due to the retrospective nature of this study and the limiter number of patients with extensive sequencing.

We showed that intensively treated patients receiving HSCT were associated with an overall favorable prognosis. Regarding the high rate of relapse in non-transplanted patients, maintenance strategies should be considered for HSCT-ineligible patients [20,21,22]. CC-486, an orally available form of azacytidine, has shown to improve OS in patients reaching CR1 after intensive chemotherapy [23]. CC-486 has been recently approved by the Food and Drug Administration (FDA) and European Medicines Agency (EMA) and might be of interest in these settings. More recently, several MLL-rearranged leukemias were shown to be dependent on the only known histone 3 lysine 79 (H3K79) methyltransferase DOT1L. In this context, Kühn et al. showed in vitro that *KMT2A-PTD*-mutated cells underwent downregulation of HOXA-cluster, and other MLL target genes followed by cell differentiation, apoptosis and cell death upon DOT1L inhibitor treatment [24]. The first pre-clinical and clinical results of DOT1L inhibitors in human settings have been encouraging so far, though they have not yet been extensively tested in *KMT2A-PTD* settings [25,26,27]. Other strategies, such as menin-KMT2A inhibitors, also exhibit high efficacy in vitro on a broad range of *KMT2A*-rearranged AML cell lines [28]. Phase 1/2a, first-in-human study of KO-539, a menin-KMT2A inhibitor is still ongoing (NCT04067336). Finally, *KMT2-PTD* AML usually expresses a specific transcriptional program, through multiple homeobox-related gene family members upregulation, closely related to LSC status [29]. Moreover, this specific genomic subgroup is frequently associated with concurrent *IDH1/2* mutations. This LSC-specific signature has been recently associated with deep and durable responses observed in newly diagnosed AML treated with the selective inhibitor of BCL-2 venetoclax in combination with azacytidine, especially in *IDH1/2*-mutated patients [30]. Overall, there might be numerous therapeutic alternatives to intensive chemotherapy for unfit patients to overcome *KMT2A-PTD* poor prognosis.

## 5. Conclusions

In conclusion, whether *KMT2A-PTD* acts as an independent prognosis marker is still rather unclear. Our results emphasize that *KMT2A-PTD* might be considered as a potential adverse prognostic factor, especially in elderly and/or with concurrent RTK/RAS pathway mutations. However, its independent prognostic value, especially within the favorable intermediate risk group and the role of upfront HSCT, should be validated in a larger prospective cohort of patient. As unfit and HSCT-ineligible patients are characterized by a very poor outcome, targeted therapies such as IDH inhibitors and venetoclax-based regimens might be of interest in this specific mutational setting.

## Figures and Tables

**Table 1:** Patient characteristics.

Variable		Total (*n* = 79)
**Age at diagnosis, median (min–max)** - **<40 years old, *n* (%)** - **40–59 years old, *n* (%)** - **>60 years old, *n* (%)**		66 (19–87) 9/79 (11.4%) 21/79 (26.6%) 49/79 (62%)
**PS ≥ 2, *n* (%)**		12/50 (24%)
**Sex ratio M/F**		1.1
**Normal karyotype, *n* (%)**		47/72 (65.3%)
**FLT3-ITD, *n* (%)**		20/70 (28.6%)
**NPM1, *n* (%)**		12/75 (16%)
**IDH1/2, *n* (%)**		20/40 (50%)
**ELN 2013 classification** - **Favorable, *n* (%)** - **Intermediate I/II, *n* (%)** - **Unfavorable, *n* (%)**		8/70 (11.4%) 54/70 (77.2%) 8/70 (11.4%)
**Secondary AML, *n* (%)**		20/79 (25.2%)
**NGS at diagnosis, *n* (%)**		24/79 (30.4%)
**Molecular subgroups**	DNMT3A, *n* (%)	12/24 (50%)
	ASXL1, *n* (%)	2/24 (8.3%)
	RUNX1, *n* (%)	6/24 (25%)
	TET2, *n* (%)	5/24 (20.8%)
	TP53, *n* (%)	1/24 (4.2%)
	SRSF2, *n* (%)	5/24 (20.8%)
	STAG2, *n* (%)	5/24 (20.8%)
	RAS/PTPN11, *n* (%)	4/24 (16.7%)

**Table 2:** Multivariate analysis

Variables	OS			LFS		
	HR (IC 95%)	Range	*p*-Value	HR (IC 95%)	Range	*p*-Value
Age < 60 vs. ≥60 years old	1.74	(0.77–3.91)	0.180	2.15	(0.99–4.67)	0.053
HSCT (yes vs. no)	2.35	(1.06–5.19)	**0.034**	2.36	(1.08–5.13)	**0.031**
*FLT3-ITD* (mut vs. wt)	0.29	(0.11–0.78)	**0.014**	0.27	(0.11–0.70)	**0.007**

Bold was used to highlight statistically significant results.

## Data Availability

Data available on request due to restrictions.

## Supplementary Materials

The following are available online at https://www.mdpi.com/article/10.3390/cancers13092272/s1, Table S1: Univariate analysis.

## References

[1] Döhner H., Estey E., Grimwade D., Amadori S., Appelbaum F.R., Büchner T., Dombret H., Ebert B.L., Fenaux P., Larson R.A. (2017). Diagnosis and management of AML in adults: 2017 ELN recommendations from an international expert panel. Blood.

[2] Huet S., Paubelle E., Lours C., Grange B., Courtois L., Chabane K., Charlot C., Mosnier I., Simonet T., Hayette S. (2018). Validation of the prognostic value of the knowledge bank approach to determine AML prognosis in real life. Blood.

[3] Papaemmanuil E., Gerstung M., Bullinger L., Gaidzik V.I., Paschka P., Roberts N.D., Potter N.E., Heuser M., Thol F., Campbell P.J. (2016). Genomic Classification and Prognosis in Acute Myeloid Leukemia. N. Engl. J. Med..

[4] Schoch C., Schnittger S., Klaus M., Kern W., Hiddemann W., Haferlach T. (2003). AML with 11q23/MLL abnormalities as defined by the WHO classification: Incidence, partner chromosomes, FAB subtype, age distribution, and prognostic impact in an unselected series of 1897 cytogenetically analyzed AML cases. Blood.

[5] Vetro C., Haferlach T., Meggendorfer M., Stengel A., Jeromin S., Kern W., Haferlach C. (2020). Cytogenetic and molecular genetic characterization of KMT2A-PTD positive acute myeloid leukemia in comparison to KMT2A-Rearranged acute myeloid leukemia. Cancer Genet..

[6] Steudel C., Wermke M., Schaich M., Schäkel U., Illmer T., Ehninger G., Thiede C. (2003). Comparative analysis of MLL partial tandem duplication and FLT3 internal tandem duplication mutations in 956 adult patients with acute myeloid leukemia. Genes Chromosom. Cancer.

[7] Schnittger S., Kinkelin U., Schoch C., Heinecke A., Haase D., Haferlach T., Büchner T., Wörmann B., Hiddemann W., Griesinger F. (2000). Screening for MLL tandem duplication in 387 unselected patients with AML identify a prognostically unfavorable subset of AML. Leukemia.

[8] Dorrance A.M., Liu S., Yuan W., Becknell B., Arnoczky K.J., Guimond M., Strout M.P., Feng L., Nakamura T., Yu L. (2006). Mll partial tandem duplication induces aberrant Hox expression in vivo via specific epigenetic alterations. J. Clin. Investig..

[9] Hinai A.S.A.A., Pratcorona M., Grob T., Kavelaars F.G., Bussaglia E., Sanders M.A., Nomdedeu J., Valk P.J.M. (2019). The landscape of KMT2A -PTD AML: Concurrent mutations, gene expression signatures, and clinical outcome. HemaSphere.

[10] Schnittger B.S., Wo B., Hiddemann W., Griesinger F. (1998). Partial Tandem Duplications of the MLL Gene Are Detectable in Peripheral Blood and Bone Marrow of Nearly All Healthy Donors. Blood.

[11] Döhner K., Tobis K., Ulrich R., Fröhling S., Benner A., Schlenk R.F., Döhner H. (2002). Prognostic Significance of Partial Tandem Duplications of the MLL Gene in Adult Patients 16 to 60 Years Old with Acute Myeloid Leukemia and Normal Cytogenetics: A Study of the Acute Myeloid Leukemia Study Group Ulm. J. Clin. Oncol..

[12] Haferlach C., Haferlach T. (2013). Another piece of the AML puzzle. Blood J. Am. Soc. Hematol..

[13] Simons A., Shaffer L.G., Hastings R.J. (2013). Cytogenetic Nomenclature: Changes in the ISCN 2013 Compared to the 2009 Edition. Cytogenet. Genome Res..

[14] Fattoum J., Cannas G., Elhamri M., Tigaud I., Plesa A., Heiblig M., Plesa C., Wattel E., Thomas X. (2015). Effect of Age on Treatment Decision-Making in Elderly Patients with Acute Myeloid Leukemia. Clin. Lymphoma Myeloma Leuk..

[15] Shih L.Y., Liang D.C., Fu J.F., Wu J.H., Wang P.N., Lin T.L., Dunn P., Kuo M.C., Tang T.C., Lin T.H. (2006). Characterization of fusion partner genes in 114 patients with de novo acute myeloid leukemia and MLL rearrangement. Leukemia.

[16] Shimada A., Taki T., Tabuchi K., Taketani T., Hanada R., Tawa A., Tsuchida M., Horibe K., Tsukimoto I., Hayashi Y. (2008). Tandem Duplications of MLL and FLT3 Are Correlated with Poor Prognoses in Pediatric Acute Myeloid Leukemia: A Study of the Japanese Childhood AML Cooperative Study Group. Pediatr. Blood Cancer.

[17] Bazarbachi A., Bug G., Baron F., Brissot E., Ciceri F., Dalle I.A., Döhner H., Esteve J., Floisand Y., Giebel S. (2020). Clinical practice recommendation on hematopoietic stem cell transplantation for acute myeloid leukemia patients with FLT3-internal tandem duplication: A position statement from the Acute Leukemia Working Party of the European Society for Blood and Marrow. Haematologica.

[18] Schlenk R.F., Kayser S., Bullinger L. (2014). Differential impact of allelic ratio and insertion site in FLT3-ITD-positive AML with respect to allogeneic transplantation. Blood.

[19] Tan Y.-T., Sun Y., Zhu S.-H., Ye L., Zhao C.-J., Zhao W.-L., Chen Z., Chen S.-J., Liu H. (2016). Deregulation of HOX genes by DNMT3A and MLL mutations converges on BMI1. Leukemia.

[20] Oran B., de Lima M., Garcia-Manero G., Thall P.F., Lin R., Popat U., Alousi M., Hosing C., Giralt S., Rondon G. (2020). A phase 3 randomized study of 5-azacitidine maintenance vs. observation after transplant in high-risk AML and MDS patients. Blood Adv..

[21] Bug G., Burchert A., Wagner E.M., Kröger N., Berg T., Güller S., Metzelder S.K., Wolf A., Hünecke S., Bader B. (2017). Phase I/II study of the deacetylase inhibitor panobinostat after allogeneic stem cell transplantation in patients with high-risk MDS or AML (PANOBEST trial). Leukemia.

[22] Oshikawa G., Kakihana K., Saito M., Aoki J., Najima Y., Kobayashi T. (2015). Post-transplant maintenance therapy with azacitidine and gemtuzumab ozogamicin for high-risk acute myeloid leukaemia. Br. J. Haematol..

[23] Wei A.H., Döhner H., Pocock C., Montesinos P., Afanasyev B., Dombret H., Ravandi F., Sayar H., Jang J.-H., Porkka K. (2020). Oral Azacitidine Maintenance Therapy for Acute Myeloid Leukemia in First Remission. N. Engl. J. Med..

[24] Kühn M.W., Hadler M.J., Daigle S.R., Koche R.P., Krivtsov A.V., Olhava E.J., Caligiuri M.A., Huang G., Bradner J.E., Pollock M. (2015). MLL partial tandem duplication leukemia cells are sensitive to small molecule DOT1L inhibition. Haematologica.

[25] Lonetti A., Indio V., Laginestra M.A., Tarantino G., Chiarini F., Astolfi A., Bertuccio S.N., Martelli A.M., Locatelli F., Pession A. (2020). Inhibition of Methyltransferase DOT1L Sensitizes to Sorafenib Treatment AML Cells Irrespective of MLL-Rearrangements: A Novel Therapeutic Strategy for Pediatric AML. Cancers.

[26] Liu W., Deng L., Song Y., Redell M. (2014). DOT1L inhibition sensitizes MLL-rearranged AML to chemotherapy. PLoS ONE.

[27] Stein E.M., Garcia-Manero G., Rizzieri D.A., Tibes R., Berdeja J.G., Savona M.R., Jongen-Lavrenic M., Altman J.K., Thomson B., Blakemore S.J. (2018). The DOT1L inhibitor pinometostat reduces H3K79 methylation and has modest clinical activity in adult acute leukemia. Blood.

[28] Brzezinka K., Nevedomskaya E., Lesche R., Steckel M., Eheim A.L., Haegebarth A., Stresemann C. (2019). Functional diversity of inhibitors tackling the differentiation blockage of MLL-rearranged leukemia. J. Hematol. Oncol..

[29] Jung N., Dai B., Gentles A.J., Majeti R., Feinberg A.P. (2015). An LSC epigenetic signature is largely mutation independent and implicates the HOXA cluster in AML pathogenesis. Nat. Commun..

[30] Pollyea D.A., Stevens B.M., Jones C.L., Winters A., Pei S., Minhajuddin M., D’Alessandro A., Culp-Hill R., Riemondy K.A., Gillen A.E. (2018). Venetoclax with azacitidine disrupts energy metabolism and targets leukemia stem cells in patients with acute myeloid leukemia. Nat. Med..

