# Supplementary material for: Allogenic Stem Cell Transplantation Abrogates Negative Impact on Outcome of AML Patients with KMT2A Partial Tandem Duplication
Source: Cancers (Basel). 2021 May 10;13(9):2272. doi: 10.3390/cancers13092272 (PMC8126020; doi:10.3390/cancers13092272)
Supplement: Supplementary file 1 [file cancers-13-02272-s001.zip › cancers-1154564-supplementary.pdf]

Supplementary Materials

Allogenic Stem Cell Transplantation Abrogates Negative Impact on Outcome of AML Patients with KMT2A Partial Tandem Duplication

Gabriel Antherieu, Audrey Bidet, Sarah Huet, Sandrine Hayette, Marina Migeon, Lisa Boureau, Pierre Sujobert, Xavier Thomas, Hervé Ghesquières, Arnaud Pigneux and Mael Heiblig

Table S1. Univariate analysis.

Variables	OS			LFS		
	**HR [IC 95%]**	**Range**	***p*-Value**	**HR [IC 95%]**	**Range**	***p*-Value**
Age < 60 vs ≥ 60 years old	0.3905	(0.19–0.82)	**0.012**	0.3336	(0.16–0.68)	**0.003**
PS ≥ 2	2.48	(1.03–5.96)	**0.042**	1.94	(0.87–4.3)	0.105
Secondary AML (yes vs no)	1.52	(0.62–3.74)	0.360	1.54	(0.65–3.63)	0.320
Karyotype (normal vs abnormal)	1.133	(0.52–2.47)	0.754	1.257	(0.59–2.67)	0.550
HSCT (yes vs no)	0.2930	(0.14–0.61)	**<0.001**	0.272	(0.14–0.55)	**<0.001**
FLT3-ITD (mut vs wt)	2.755	(1.08–7.00)	**0.033**	2.641	(1.05–6.64)	**0.038**
NPM1 (mut vs wt)	0.5063	(0.19–1.37)	0.179	0.66	(0.24–1.76)	0.440

Bold refers to statistically significant results.

© 2021 by the authors. Licensee MDPI, Basel, Switzerland. This article is an open access article distributed under the terms and conditions of the Creative Commons Attribution (CC BY) license (http://creativecommons.org/licenses/by/4.0/).
